# Draft Genome Sequence of Plant Growth-Promoting Drought-Tolerant *Bacillus* sp. Strain CMAA 1363 Isolated from the Brazilian Caatinga Biome

**DOI:** 10.1128/genomeA.01534-16

**Published:** 2017-02-02

**Authors:** Vanessa Nessner Kavamura, Suikinai Nobre Santos, Rodrigo Gouvêa Taketani, Rafael Leandro Figueiredo Vasconcellos, Itamar Soares Melo

**Affiliations:** Laboratory of Environmental Microbiology, Embrapa Environment, Jaguariúna, São Paulo, Brazil

## Abstract

The strain of *Bacillus* sp. CMAA 1363 was isolated from the Brazilian Caatinga biome and showed plant growth-promoting traits and ability to promote maize growth under drought stress. Sequencing revealed genes involved in stress response and plant growth promotion. These genomic features might aid in the protection of plants against the negative effects imposed by drought.

## GENOME ANNOUNCEMENT

Strain CMAA 1363 was originally recovered from the rhizosphere of *Cereus jamacaru*, a cactus found in a unique Brazilian semiarid biome called Caatinga (3 to 17°S to 35 to 45°W). This strain showed plant growth-promoting traits and the ability to promote maize growth under drought stress (1), and it displayed 99.6% 16S rRNA gene similarity to *Bacillus aryabhattai*, a Gram-positive bacterium that has been recently described by Shivaji et al. (2). Genomic DNA was extracted from a pure culture grown overnight on tryptic soy broth (TSB) medium using the PureLink genomic kit (Life Technologies, Inc.). Whole-genome sequencing was performed using the Ion Torrent (PGM) platform, according to the manufacturer’s protocol. Genome sequence was *de novo* assembled using MIRA version 4, CLC Genomics Workbench version 5.5.1, and SeqMan NGen version 4.0.0 packages. The obtained contigs were integrated using CISA, according to Santos et al. (3).

The genome size corresponded to 3,656,253 bp, with 135× coverage. Data were assembled using *Bacillus aryabhattai* strain T61 (GenBank accession no. NZ_KQ087173) (Yan et al. [4]) as a reference and analyzed by Rapid Annotations using Subsystems Technology (RAST) (5). The genome size was found to be 4,956,314 bp, allocated into 10 contigs, comprising 5,339 coding sequences, 414 subsystems, and 63 RNA genes. A total of 126 genes involved in stress response were found. Forty-one genes involved in cell wall and capsule were found, with seven of them being related to capsular and extracellular polysaccharide biosynthesis. Also, six genes related to auxin biosynthesis and 529 genes related to amino acids and derivatives were found. As previously mentioned, the closest species is *Bacillus aryabhattai*, which has been reported as temperature and drought tolerant (6), and its genome revealed adaptations to the Tibetan plateau, which has high altitude, high UV radiation, and limited oxygen and temperature variations (4). Some of these characteristics (high UV radiation and temperature variation) may be similar to those found in the Caatinga biome, enabling the survival of this strain under such conditions. Furthermore, some genomic features, such as amino acids, exopolysaccharides, and indole-3-acetic acid (IAA) biosynthesis, might aid in plant growth promotion and protection against negative effects imposed by drought.

### Accession number(s).

The partial genome sequence of *Bacillus* sp. CMAA 1363 has been ascribed to the whole-genome shotgun project deposited at DDBJ/ENA/GenBank under the accession no. MJGZ00000000. The version described in this paper is version MJGZ01000000.
